# Can 3D-printing avoid discomfort-related implant removal in midshaft clavicle fractures? A four-year follow-up

**DOI:** 10.1007/s00402-020-03654-6

**Published:** 2020-10-31

**Authors:** Rob F. M. van Doremalen, Rens A. van der Linde, Jan J. Kootstra, Sven H. van Helden, Edsko E. G. Hekman

**Affiliations:** 1 Department of Surgery, Isala Hospital, 8000 GK Zwolle, The Netherlands; 2 Department of Robotics and Mechatronics, Faculty of Electrical Engineering, Mathematics and Computer Science, University of Twente, P.O. Box 217, 7500 AE Enschede, The Netherlands; 3 Department of Biomechanical Engineering, University of Twente, 7500 AE Enschede, The Netherlands; 4 Deventer Hospital, 7400 GC Deventer, The Netherlands; 5 Department of Surgery, Medisch Spectrum Twente, 7500 KA Enschede, The Netherlands; 6 Department of Surgery, Hospital Group Twente, 7600 SZ Almelo, The Netherlands

**Keywords:** Midshaft clavicle fracture, Plate fixation, Preoperative preparation, 3D-printing, Reoperation rate

## Abstract

**Introduction:**

Due to the variation in shape and curvature of the clavicle, plates often have to be adjusted during surgery to acquire a good fit. Poorly fitted plates can cause discomfort, eventually requiring implant removal. 3D-printed replicas of the fractured clavicle can assist in planning of the surgical approach, plate selection and, if necessary, adjustment of the plate prior to surgery. We hypothesized this method of preoperative preparation would reduce implant-related discomfort resulting in a reduced reoperation rate

**Materials and methods:**

In a prospective cohort study, perioperative plate handling and clavicle fixation were timed and follow-up data were collected from participants undergoing operative treatment for a midshaft clavicle fracture. The control group (*n* = 7) received conventional surgery with standard precontoured plates. For the intervention group (*n* = 7), 3D-printed replicas of the fractured clavicle and a mirrored version of the healthy contralateral clavicle were available prior to surgery for planning of the surgical approach, and for plate selection and contouring. Primary outcome was reoperation rate due to implant-related discomfort. Secondary outcomes were complications and time differences in the different surgical phases (reduction, fixation and overall operation time)

**Results:**

More participants in the control group had the plate removed due to discomfort compared to the intervention group (5/7 vs. 0/6; *P* = 0.012). One participant was excluded from the intervention group due to a postoperative complication; an infection occurred at the implant site. No relevant time difference in surgical plate handling was found between both groups.

**Conclusions:**

Preoperative preparation using 3D-printed replicas of the clavicle fracture may reduce implant removal caused by plated-related discomfort. No relevant effect on surgery time was found.

**Trial registration:**

Registered with ‘toetsingonline.nl’, trial number NL51269.075/14, 17-02-2015

## Introduction

Clavicle fractures account for 2–5% of all clinically presented fractures in adults [1–3]. Most fractures (81%) are located in the midshaft or diaphysis of the clavicle (type 15-B as classified by the Orthopaedic Trauma Association) [4]. Conservative treatment, by mere immobilization of the shoulder with a sling, is sufficient in the majority of fractures. However, more severely displaced fractures may require surgical intervention [1, 5–8].

Surgical treatment often consists of open reduction and internal fixation (ORIF) with plates [9]. Different types of precontoured clavicle plates are available, but a good fit is often difficult to acquire due to variations in shape and curvature of the clavicle [10]. Plates are selected and manually adjusted intra-operatively to follow the curvature of the clavicle to prevent excessive space between the clavicle and plate, thereby achieving rigid fixation and minimizing patient discomfort. However, the sterile conditions in the operating theater are suboptimal for these activities, due to limited view and access to the fracture. Plate-related discomfort is one of the main reasons for implant removal in a secondary operation. In 20–39 percent of the cases, the implant is removed within three years [11–14]. Using patient-specific 3D-printed replicas of the fractured and the contralateral clavicles, it is possible to select, pre-contour and pre-fit a suitable plate pre-operatively [15]. Also, the approach for reduction and fixation can be planned ahead.

The aim of this study was to test if surgical preparation using 3D-printed replicas can improve surgical midshaft clavicle fracture treatment. Similar methods have been described for minimal invasive fixation of clavicle fractures and acetabular and calcaneal fractures, with promising results [16–20]. We hypothesized that, using this technique, patient discomfort would decrease resulting in fewer secondary interventions for implant removal. Furthermore, we hypothesized a reduction in both operation time and complications could be achieved.

## Materials and methods

### Study design

This study was designed as a single-center, non-blinded, prospective cohort clinical trial. The study was reviewed and approved by the local institutional review board (protocol 14.11157l) and registered with ‘toetsingonline.nl’ (#NL51269.075/14). All participants provided written informed consent prior to enrollment in the study.

Participants were included from the outpatient trauma clinic of a large regional hospital, which is also one of the major trauma centers in the Netherlands. All consecutive patients between October 2014 and June 2015, with an acute midshaft clavicle fracture who considered operative treatment, were asked to participate. All procedures were performed by experienced trauma surgeons and/or final-year trauma residents. Furthermore, participants were followed for a total of four years.

### Inclusion and exclusion criteria

The inclusion criteria for this study were: all midshaft clavicle fractures (OTA type 15-B), with an age between seventeen and seventy, and an indication for ORIF. Indications for ORIF included: displacement or shortening of the clavicle, neurovascular compromise, open fractures, superior displacement of fracture elements with tenting of the skin or expected patient benefit from a quick recovery. Exclusion criteria were: non-midshaft fractures, an age outside the seventeen to seventy range, pregnancy, and a history of one, or multiple clavicle fractures. Patients with insufficient understanding of the Dutch language or deemed incapable of participation were also excluded.

### 3D-printing patient-specific plastic replicas

For this study, we 3D-printed a replica of the fractured clavicle and healthy reconstruction of the fractured clavicle by mirroring the contralateral healthy clavicle. The process of producing these replicas consisted of three consecutive steps: (1) imaging, (2) image processing and (3) 3D-printing.

**Fig. 1:**
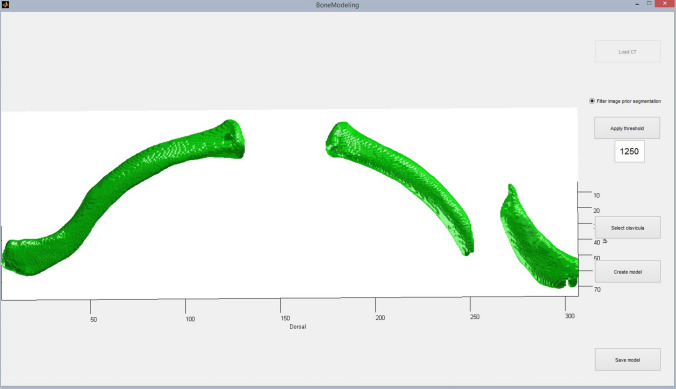
Screenshot of three-dimensional surface mesh models of the fractured clavicle (right) and the contralateral clavicle (left)

**Fig. 2:**
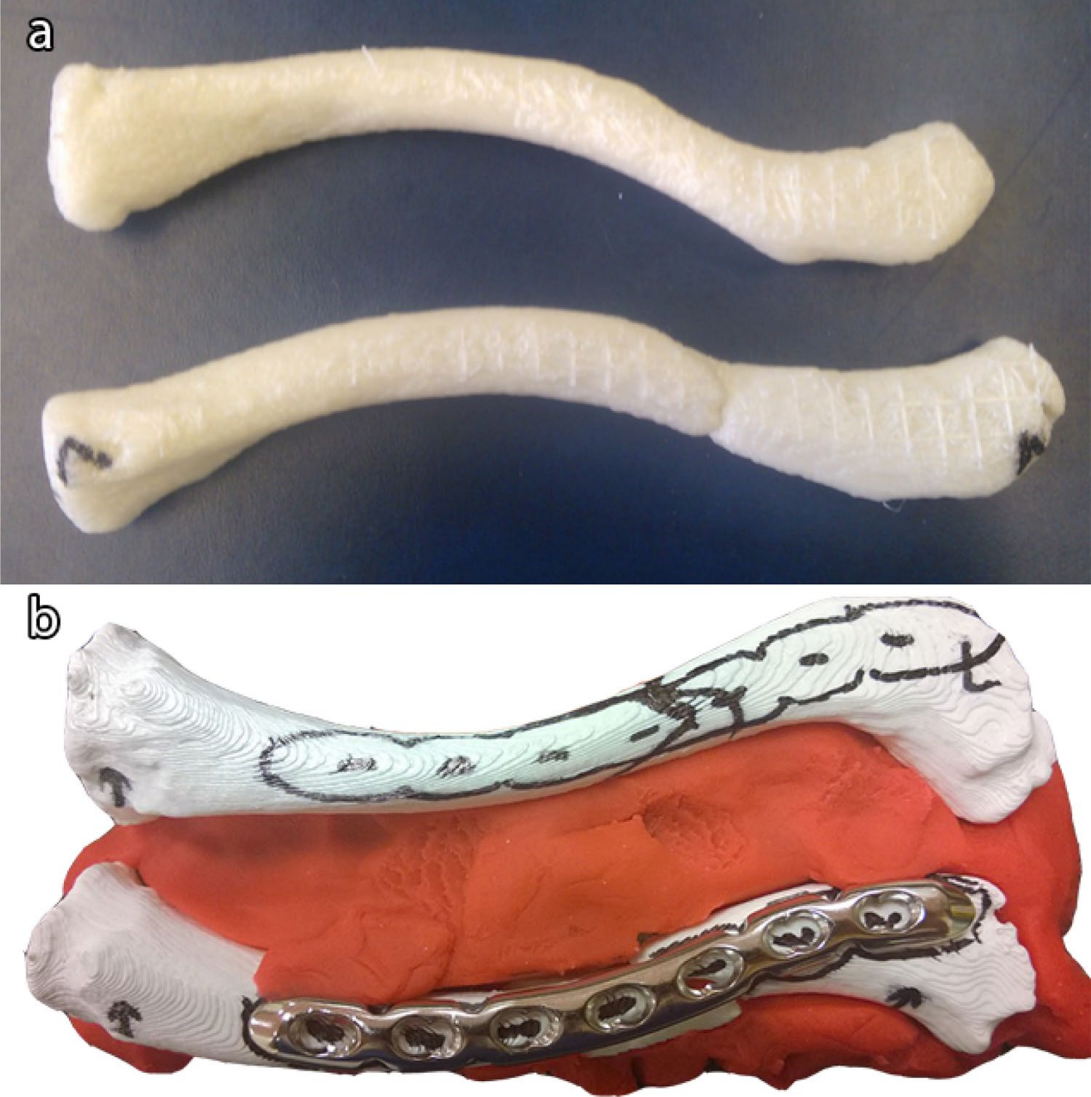
**a** 3D-Printed models of the mirrored contralateral clavicle (top) and the fractured clavicle (bottom) and **b** two models from another case are stabilized in clay for support of the fragments. The precontoured plate is outlined on both clavicles and fitted on the fractured model (bottom) and the fracture lines are marked on the mirrored contralateral clavicle (top). By impressing the mirrored contralateral clavicle in the clay, a mold is formed to together the fractured clavicle

In case no computed tomography (CT) scan was available, a low-dose CT scan of both clavicles was made at least two days prior to surgery (estimated exposure of 135 Dose-Length Product). No extra radiation was used for both clavicles in contrast to one as both are situated in the same axial slice. Both clavicles were segmented from these images, then converted in a 3D surface model and exported in the STL format using the mathematical programming software MATLAB (The Mathworks Inc. Natick, MA; Fig. 1). Through further processing in MeshLab (Visual Computing Lab, Pisa, Italy), the fractured elements were separated. Furthermore, the healthy contralateral clavicle was mirrored, using the symmetrical features of the body to create a healthy reconstruction of the fractured clavicle [21–23]. These models were 3D-printed with polylactic acid (PLA) plastic (Fig. 2a), using slicing software Cura (Version 15.02.1, Ultimaking Ltd., Geldermalsen, The Netherlands) on a BQ Witbox 3D-printer (BQ, Navarra, Spain). This fabrication process has been described in detail in literature [24–28].

The resulting 3D-printed replicas of the fractured clavicle and healthy reconstruction were used for the preparation and planning of the surgical treatment.

### Surgical preparation and planning

With the 3D-printed replicas, it is possible to plan the reposition of the fractured elements, select the most suitable plate, make necessary adjustments the shape of the plate and plan the position of the plate over the fractured clavicle.

**Fig. 3:**
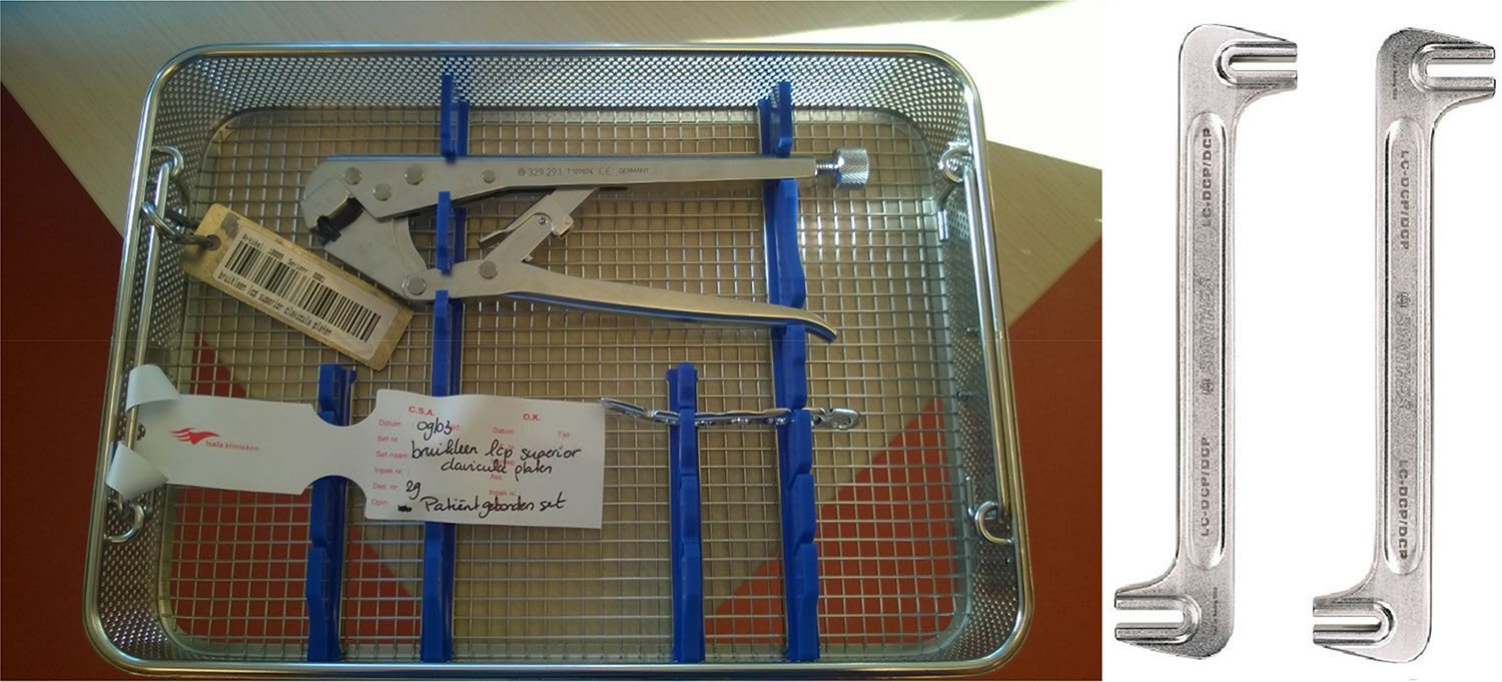
The left picture shows a pair of bending pliers and a precontoured plate in a basket, ready for sterilization. The right picture shows regular bending irons, used for both preoperative planning and perioperative plate adjustments

For support, both models were partially embedded in clay (Fig. 2b). From here, the optimal plate type and length could be determined using several different non-sterile fitting plates (3.5 mm LCP Superior Anterior Clavicle Plates, Synthes Depuy). Criteria for a good fit included: optimal surface conformity, and a minimum of two, preferably three, bicortical screws in both the main proximal and distal fragment. If a standard plate did not fit well enough adjustments were made using appropriate surgical tools (Synthes Depuy; Fig. 3) and the plate would be resterilized prior to surgery. The position of the plate and proposed surgical approach were discussed with the surgeons and documented before surgery.

### Operative intervention

Standard hospital protocols were retained, to minimize differences in surgical procedures between the control- and intervention group. Both groups received prophylactic antibiotics and underwent superior plate fixation of the injured clavicle, followed by control fluoroscopy prior to wound closure. In literature, this surgical technique has been described in detail before [29]. All participants were provided with a sling for the first postoperative week, after which small range-of-motion exercises were introduced. Follow-up appointments were made at two and six weeks after surgery, for purposes of wound inspection and function evaluation, respectively. In addition, participants were provided with rehabilitation instructions.

### Outcome measures

The primary outcome was time to reinterventions for discomfort-related implant removal, for which patients were monitored in a four-year follow-up. Plate discomfort can be caused by plate design, positioning and fit. Plate design and positioning remained constant, only fit was altered with our proposed preparation method. Secondary outcomes were: complications and relevant time difference in surgical plate handling. The plate handling time was isolated from the surgery time with perioperative time measurements.

Five different moments were defined (T1–T5).

- T1 Incision
- T2 Start of reduction: the fracture site has been exposed and surgeons make a first attempt to reduce the fracture.
- T3 Plate fixation: the first screw is placed.
- T4 The last screw is put into place.
- T5 The wound is closed.

T1–T5 defines the standard overall surgery time. The period between T2 and T4 represents the time needed for the entire process of plate fixation and specifically T2 till T3 was the process of fracture reduction. Time differences larger than 5 min were considered to be relevant.

### Statistical analysis

Statistical analysis was performed using SPSS software (Version 22, IBM corp **©**, Arkmonk, New York, United States). Descriptive statistics were used to describe patient characteristics of the different groups. For the primary outcome, a Kaplan–Meier curve with Log Rank (Mantel–Cox) test was performed. For the secondary outcome, the student *t* test was used to compare the means of parametric independent variables. Non-parametric variables were assessed using a Mann–Whitney *U* test. Results with *P* < 0.05 were considered to be significant.

## Results

### Study population

**Fig. 4:**
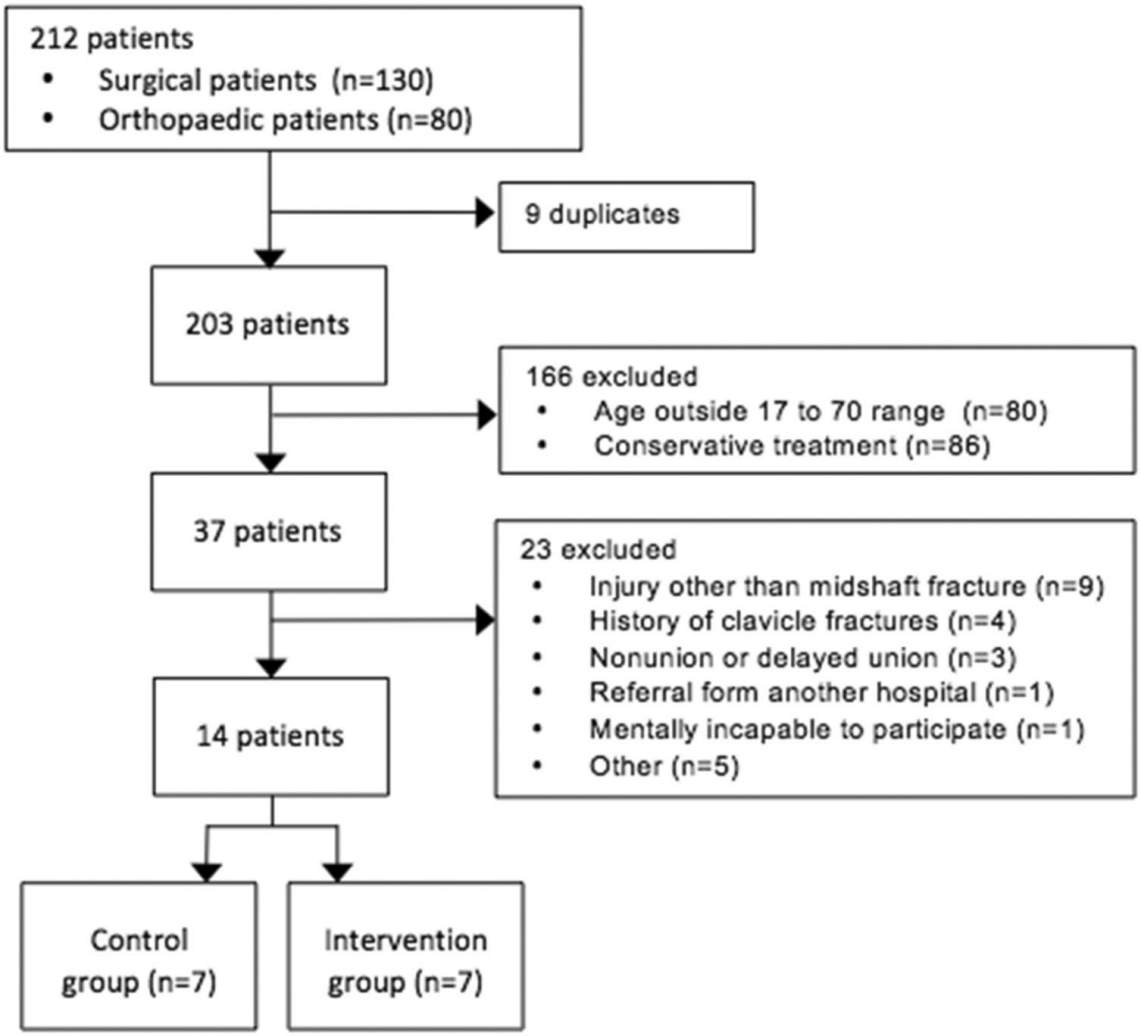
Flowchart of the patient selection procedure

Two hundred and three patients presented at our hospital between October 2014 and June 2015, with a clavicle fracture. A total of 189 patients were excluded (Fig. 4), the remaining fourteen patients, with an equal number of fractures, participated in the study. Patient characteristics are reported in Table 1.

**Table 1:** Patient characteristics

Characteristics	Control group (*n* = 7)	Intervention group (*n* = 7)	P*
Sex			
Male	6	6	1.000
Female	1	1	1.000
Age (SD)	36.6 (20.8)	42.0 (12.9)	0.571
Fracture characteristics			
Days between injury and surgery (SD)	12.0 (2.8)	16.6 (6.5)	0.127
Dislocation	7	6	0.299
Shortening	2	3	0.577
Comminution	4	5	0.577
OTA classification (B1/B2)	3/4	2/5	0.577

*Differences were considered significant at *P* < 0.05

### Discomfort-related implant removal

Compared to the intervention group, participants in the control group underwent significantly more reoperations for removal of the plate (5/7 vs. 0/7; *P* = 0.012). Five participants (71.5%) in the control group experienced plate-related discomfort requiring implant removal. No participants in the intervention group suffered from plate-related discomfort requiring implant removal. However, one participant from the intervention group was excluded due to an infection complication (5/7 vs. 0/6; *P* = 0.012).

### Perioperative time measurements

Perioperative time measurements are reported in Table 2. Only plate fixation time had a significant and relevant time difference (> 5 min) in favor of the control group (6:57 min; *P* = 0.032).

**Table 2:** Time measurements^a^

Phase	Control group (*n* = 7)	Intervention group (*n* = 7)	Δ time	*P* value
Reduction (T2–T3)	21:32 (7:12)	18:10 (8:12)	3:22	0.430
Fixation (T3–T4)	08:55 (1:15)	15:52 (6:37)	− 6:57	0.032*
Fracture repair (T2–T4)	30:28 (7:09)	34:03 (9:45)	− 3:25	0.450
Operation time (T1–T5)	54:25 (10:27)	1:02:08 (12:07)	− 7:42	0.227

^a^All measurements are noted as minutes and seconds (mm:ss), or hours, minutes and seconds (h:mm:ss) with the standard deviation in parenthesis

*Differences were considered significant at *P* < 0.05

### Complications

In the intervention group, one patient (14.3%) suffered from a delayed union twelve weeks after the initial procedure due to an infection at the implant site. The patient was treated by surgical debridement of the wound and refixation with a standard plate.

## Discussion

### Introduction

The aim of this study was to investigate the patient benefit from preoperative preparation using 3D-printed replicas of the fractured clavicle and a healthy mirrored reconstruction. We compared two patient groups, each with seven participants who underwent plate fixation for an acute midshaft clavicle fracture. The control group was treated with conventional plate fixation using standard precontoured plates, the intervention group was treated using the preoperative preparation as described. The results showed a significant difference in discomfort-related implant removal in favor of the intervention group, which may indicate that preoperative preparation could reduce plate-related discomfort. Other than the fixation time, no relevant perioperative time differences were observed. With one infection (14%) at the implant site in the intervention group, there was no significant difference in complication rate.

### Discussion results

Our results indicate that preoperative preparation using 3D-printed replicas of the clavicle could result in fewer discomfort-related secondary operations. Assuming that a poorly fitted plate is one of the main reasons for discomfort in patients, ensuring a good fit may spare patients a secondary operation for implant removal. Every surgery holds a risk, is expensive and is a burden on the patient, so avoiding secondary surgery has a positive impact on the patient and healthcare expenses. As for perioperative time differences, we expected a shortening because the plate selection and adjustment steps were taken out of the operating room. However, the only significant time difference was found in the fixation phase, and in favor of the control group. The reason for this contradiction can probably be explained by the apparent learning curve of the procedure. The procedure was new for all participating physicians and the seven cases were distributed over five surgeons, so no data beyond the learning curve could be perceived. Last, only one complication was registered, a delayed union due to infection in the intervention group.

### Research design

This study has several strengths and limitations. The most important limitation is the small number of participants, which makes it difficult to draw any definitive conclusions. However, this study has a long follow-up time; all patients were followed for a minimum of four years. According to a large retrospective cohort within our hospital, 99% of the implant removal is performed within three years after surgery, so we can assume that the chance of missing any secondary interventions is small. Second, selection of participating surgeon was not altered from daily practice. As a consequence, five different surgeons operated in the intervention group; hence, no learning curve could be studied. However, one surgeon performed three operations in the intervention group within a short period of time and we noticed that he slowly learned to adapt the procedure to his advantage, which may indicate that more benefit could be gained. Besides, the heterogeneity of the participating surgeons has a limiting effect on comparing surgical time in this small cohort. Last, the control group has a high reoperation rate (71%) compared to a retrospective cohort in our hospital (39%) [14]. All time measurements were executed by the coordinating investigator to ensure consistency.

### Method evaluation

A CT scan protocol was designed with the lowest radiation dose possible, while preserving bone tissue contrast for segmentation according to the ALARA (as low as reasonably possible) principle. Given the potential benefit of reduced reoperation rate, the extra radiation rate is deemed justifiable. In two cases (29%), a suitable CT scan was available and therefore, the subjects were not exposed to extra radiation for this study. No extra radiation was used for imaging both clavicles as opposed to one, because both are in the same transversal plane.

During the course of this study, the time it took to prepare the 3D models for 3D-printing was reduced from four hours to one hour. For implementation in daily practice, this process can be optimized and shortened even further. Recent developments enable clinical staff to do the preprocessing within the hospital IT environment with dedicated software linked to the PACS. 3D-printing time itself can also be optimized. We managed to print both replicas in around three hours; however by, for instance, increasing the nozzle size, this can be done in less time. By increasing nozzle size, some submillimeter detail will be lost, which is not required for this application.

Not all plates need adjustment preoperatively. While planning placement of the plate, the position of the surgical reposition clamps for temporary fixation is taken into account. Perioperatively plate adjustment was documented in 6 out of 7 cases in the control group and one case in the intervention group. The latter was caused by an obstructing lag screw to fixate the acquired reduction, this emphasizes the importance of planning and discussing the temporal fixation of the reduction before plate adjustment.

### Future perspective

Even though it was not in the scope of this study, some extra benefits were observed and discussed. As one surgeon performed three consecutive surgeries in the intervention group, he gained confidence in the shape of the plate cohering to the patient’s clavicle and in the planned placement of the plate related to the fracture lines. With enough confidence in a good plate fitting, the cortex can be exposed without visible conformation of reposition, which makes it possible to shorten the needed incision. This can increase esthetic outcome and possibly reduce loss of sensation in the skin. In addition, with this confidence, the plate can be used to guide the reduction of the fracture. Based on the fracture line, the plate can be placed and fixated laterally and subsequently medially, by temporarily clamping the plate to the clavicle. This way the placement of the plate is not hindered by the reposition clamps, which are normally placed to temporarily hold the reduction, and the most ideal placement can be used.

Furthermore, having the plastic replicas and a digital representation of inpatient orientation available in the OR proved to be a valuable tool in planning the approach and preserving bone marrow and blood supply to the cortex.

In summary, the results of this study suggest that surgical preparation with 3D-printing could lead to a reduction in discomfort-related implant removal, and related risks and expenses. Limitations are the extra work and time needed for preparation, costs, extra radiation exposure in most cases and additional sterilization before surgery. However, the current study population is too small to draw any definitive conclusions. Since the start of this study, we have seen the acceptance and integration of 3D-printing in hospitals grow. With this growth, the limitations of 3D-printing keep decreasing.

We hypothesized that the low reoperation rate in the intervention group was due to a good fit of the plate, theoretically because it improves the length, alignment and rotation of the clavicle and thereby the accuracy of the reduction. One could argue that more might be gained by applying this kind of preparation to other types of fractures, such as intra-articular fractures and fractures which are not easily accessible. Intra-articular fractures depend heavily on an accurate reduction to preserve functional outcome of the joint. In case of pelvic fractures, surgeons often experience difficulties gaining good access to the fracture, so being able to trust the shape and planned placement of the plate could be a big help.

## Conclusion

We have tested a preoperative preparation method for internal fixation of midshaft clavicle fractures using 3D-printed replicas of the clavicle fracture. Our results suggest that preoperative planning may reduce discomfort-related implant removal. This in turn leads to reduced healthcare costs. No relevant effect on surgery time was observed.

## References

[1] Postacchini F, Gumina S, De Santis P, Albo F (2002). Epidemiology of clavicle fractures. J Shoulder Elbow Surg.

[2] Neer CS (1960). Nonunion of the clavicle. J Am Med Assoc.

[3] Nordqvist A, Petersson C (1994) The incidence of fractures of the clavicle. Clin Orthop Relat Res 127–32 8131324

[4] Marsh JL, Slongo TF, Agel J, Broderick JS, Creevey W, DeCoster TA (2007). Fracture and dislocation classification compendium—2007: orthopaedic trauma association classification, database and outcomes committee. J Orthop Trauma.

[5] Virtanen K, Remes V, Malmivaara A, Paavola M (2009). Treatment of clavicle fractures: systematic review. Suomen Ortopedia Traumatol.

[6] Melenevsky Y, Yablon CM, Ramappa A, Hochman MG (2011). Clavicle and acromioclavicular joint injuries: a review of imaging, treatment, and complications. Skeletal Radiol.

[7] Wijdicks F-JG, Van der Meijden OAJ, Millett PJ, Verleisdonk EJMM, Houwert RM (2012). Systematic review of the complications of plate fixation of clavicle fractures. Arch Orthop Trauma Surg.

[8] Althausen PL, Shannon S, Lu M, O’Mara TJ, Bray TJ (2013). Clinical and financial comparison of operative and nonoperative treatment of displaced clavicle fractures. J Shoulder Elbow Surg.

[9] van der Meijden OA, Gaskill TR, Millett PJ (2012). Treatment of clavicle fractures: current concepts review. J Shoulder Elbow Surg.

[10] Vancleef S, Herteleer M, Carette Y, Herijgers P, Duflou JR, Nijs S (2019). Why off-the-shelf clavicle plates rarely fit: anatomic analysis of the clavicle through statistical shape modeling. J Shoulder Elbow Surg.

[11] Leroux T, Wasserstein D, Henry P, Khoshbin A, Dwyer T, Ogilvie-Harris D (2014). Rate of and risk factors for reoperations after open reduction and internal fixation of midshaft clavicle fractures: a population-based study in Ontario, Canada. J Bone Joint Surg Am.

[12] Fridberg M, Ban I, Issa Z, Krasheninnikoff M, Troelsen A (2013). Locking plate osteosynthesis of clavicle fractures: complication and reoperation rates in one hundred and five consecutive cases. Int Orthop.

[13] Ashman BD, Slobogean GP, Stone TB, Viskontas DG, Moola FO, Perey BH (2014). Reoperation following open reduction and plate fixation of displaced mid-shaft clavicle fractures. Injury.

[14] van der Linde RA, Beetz I, van Helden SH (2017). Plating for midshaft clavicular fractures: the impact on quality of life and functional outcome. Injury.

[15] van Doremalen RFM, Kootstra JJ, Hekman EEG, van Helden SH (2016). Use of rapid prototyping in a midschaft clavicular pseudarthrosis repair. J Shoulder Elbow Surg.

[16] Chung KJ, Hong DY, Kim YT, Yang I, Park YW, Kim HN (2014). Preshaping plates for minimally invasive fixation of calcaneal fractures using a real-size 3D-printed model as a preoperative and intraoperative tool. Foot Ankle Int.

[17] Jeong H-S, Park K-J, Kil K-M, Chong S, Eun H-J, Lee T-S (2014). Minimally invasive plate osteosynthesis using 3D printing for shaft fractures of clavicles: technical note. Arch Orthop Trauma Surg.

[18] Kim HN, Liu XN, Noh KC (2015). Use of a real-size 3D-printed model as a preoperative and intraoperative tool for minimally invasive plating of comminuted midshaft clavicle fractures. J Orthop Surg Res.

[19] Vlachopoulos L, Schweizer A, Meyer DC, Gerber C, Fürnstahl P (2017). Computer-assisted planning and patient-specific guides for the treatment of midshaft clavicle malunions. J Shoulder Elbow Surg.

[20] Kim JW, Lee Y, Seo J, Park JH, Seo YM, Kim SS (2018). Clinical experience with three-dimensional printing techniques in orthopedic trauma. J Orthop Sci.

[21] Cunningham BP, McLaren A, Richardson M, McLemore R (2013). Clavicular length: the assumption of symmetry. Orthopedics.

[22] Hingsammer AM, Lazaros V, Dominik MC, Furnstahl P (2015). Three-dimensional corrective osteotomies of mal-united clavicles—is the contralateral anatomy a reliable template for reconstruction?. Clin Anat.

[23] Grewal S, Dobbe JG, Kloen P (2018). Corrective osteotomy in symptomatic clavicular malunion using computer-assisted 3-D planning and patient-specific surgical guides. J Orthop.

[24] Brown GA, Firoozbakhsh K, DeCoster TA, Reyna JR, Moneim M (2003). Rapid prototyping: the future of trauma surgery?. J Bone Joint Surg Am.

[25] Rengier F, Mehndiratta A, Von Tengg-Kobligk H, Zechmann CM, Unterhinninghofen R, Kauczor HU, Giesel FL (2010). 3D printing based on imaging data: review of medical applications. Int J Comput Assist Radiol Surg.

[26] Bagaria V, Deshpande S, Rasalkar DD, Kuthe A, Paunipagar BK (2011). Use of rapid prototyping and three-dimensional reconstruction modeling in the management of complex fractures. Eur J Radiol.

[27] Biglino G, Schievano S, Taylor AM (2011). The use of rapid prototyping in clinical applications. Adv Appl Rapid Prototyp Technol Modern Eng.

[28] Frame M, Huntley JS (2012). Rapid prototyping in orthopaedic surgery: a user’s guide. ScientificWorldJournal.

[29] Altamimi SA, McKee MD (2008). Nonoperative treatment compared with plate fixation of displaced midshaft clavicular fractures: surgical technique. J Bone Joint Surg Am.

